# Postconcussive Symptoms After Early Childhood Concussion

**DOI:** 10.1001/jamanetworkopen.2024.3182

**Published:** 2024-03-21

**Authors:** Dominique Dupont, Ken Tang, Cindy Beaudoin, Fanny Dégeilh, Isabelle Gagnon, Keith Owen Yeates, Sean C. Rose, Jocelyn Gravel, Brett Burstein, Antonia S. Stang, Rachel M. Stanley, Roger L. Zemek, Miriam H. Beauchamp

**Affiliations:** 1 Department of Psychology, Université de Montréal, Montreal, Quebec, Canada; 2 Centre de recherche Azrieli du Centre Hospitalier Universitaire Sainte-Justine, Montreal, Quebec, Canada; 3 Independent Statistical Consultant, Richmond, British Columbia; 4 Univ Rennes, CNRS (Centre national de recherche scientifique), Inria, Inserm, IRISA (Institut de recherche en informatique et systèmes aléatoires) UMR (Unité mixte de recherche) 6074, EMPENN — ERL (Equipe de recherche labellisée) U1228, Rennes, France; 5 Montreal Children’s Hospital, McGill University Health Centre, Montreal, Quebec, Canada; 6 School of Physical and Occupational Therapy, McGill University, Montreal, Quebec, Canada; 7 Hotchkiss Brain Institute, Alberta Children’s Hospital Research Institute, Calgary, Alberta, Canada; 8 Department of Psychology, University of Calgary, Calgary, Alberta, Canada; 9 Child Neurology, Nationwide Children’s Hospital, Columbus, Ohio; 10 The Ohio State University College of Medicine Columbus, Columbus; 11 Montreal Children’s Hospital, Division of Pediatric Emergency Medicine, McGill University Health Centre, Montreal, Quebec, Canada; 12 Department of Biostatistics, Epidemiology and Occupational Health, McGill University, Montreal, Quebec, Canada; 13 Department of Pediatrics, University of Calgary, Calgary Alberta, Canada; 14 Emergency Medicine, Nationwide Children’s Hospital, Columbus, Ohio; 15 Departments of Pediatrics and Emergency Medicine, University of Ottawa, Ontario, Canada

## Abstract

**Importance:**

Research on postconcussive symptoms (PCS) following early childhood concussion has been hindered by a lack of measures suitable for this age group, resulting in a limited understanding of their evolution in young children.

**Objective:**

To document PCS in the first 3 months after early childhood concussion using a developmentally appropriate measure.

**Design, Setting, and Participants:**

This cohort study used data collected at 3 Canadian and 1 US urban pediatric emergency departments (EDs) and 8 Canadian daycares from December 2018 to December 2022 as part of the Kids’ Outcomes and Long-Term Abilities (KOALA) project, a prospective, multicenter, longitudinal cohort study. Participants included children aged 6 to 72 months with early childhood concussion or orthopedic injury (OI) or uninjured children from the community to serve as controls. Data were analyzed from March 2023 to January 2024.

**Exposure:**

Concussion sustained between ages 6 and 72 months.

**Main Outcomes and Measures:**

Primary outcomes were cognitive, physical, behavioral and total PCS assessed prior to injury (retrospectively), acutely (within 48 hours), and at 10 days, 1 month, and 3 months after injury or recruitment through caregiver observations using the Report of Early Childhood Traumatic Injury Observations & Symptoms inventory. Group comparisons were analyzed using ordinal regression models.

**Results:**

The study included 303 children (mean [SD] age, 35.8 [20.2] months; 152 [50.2%] male). Of these, 174 children had a concussion (mean [SD] age,  33.3 [19.9] months), 60 children had an OI (mean [SD] age, 38.4 [19.8] months) and 69 children were uninjured controls (mean [SD] age, 39.7 [20.8] months). No meaningful differences were found between the concussion and comparison groups in retrospective preinjury PCS. Significant group differences were found for total PCS at the initial ED visit (concussion vs OI: odds ratio [OR], 4.33 [95% CI, 2.44-7.69]; concussion vs control: OR, 7.28 [95% CI, 3.80-13.93]), 10 days (concussion vs OI: OR, 4.44 [95% CI, 2.17-9.06]; concussion vs control: OR, 5.94 [95% CI, 3.22-10.94]), 1 month (concussion vs OI: OR, 2.70 [95% CI, 1.56-4.68]; concussion vs control: OR, 4.32 [95% CI, 2.36-7.92]), and 3 months (concussion vs OI: OR, 2.61 [95% CI, 1.30-5.25]; concussion vs control: OR, 2.40 [95% CI, 1.36-4.24]). Significant group differences were also found for domain-level scores (cognitive, physical, behavioral) at various time points.

**Conclusions and Relevance:**

In this early childhood cohort study, concussion was associated with more PCS than OIs or typical development up to 3 months after injury. Given the limited verbal and cognitive abilities typical of early childhood, using developmentally appropriate manifestations and behaviors is a valuable way of tracking PCS and could aid in concussion diagnosis in young children.

## Key Points

**Question:**

What amount and type of postconcussive symptoms occur after early childhood concussion and how do they evolve?

**Findings:**

In this cohort study of 303 children aged 6 to 72 months recruited from urban pediatric emergency departments and daycares, those with concussion exhibited more postconcussive symptoms than uninjured children and children with orthopedic injury acutely and at 10 days, 1 month, and 3 months after injury. Symptoms, documented using a developmentally-appropriate measure, were most common in the physical and behavioral domains.

**Meaning:**

These findings suggest that early childhood concussion can result in significantly elevated postconcussive symptoms, which may remain present 3 months after injury and are not solely attributable to general injury effects or typical development.

## Teaser

This cohort study examines postconcussive symptoms in the first 3 months after concussion among children aged 6 to 72 months using a developmentally appropriate measure.

## Introduction

Concussions and mild traumatic brain injury (mTBI) are common during early childhood; and, in particular, children aged 5 years and younger have the highest incidence of presentation to emergency departments (EDs) among pediatric groups for injury caused by a direct or indirect force to the head.^1,2^ In the United States, more than 300 000 children present to the ED annually with these types of injuries.^3^ Postconcussive symptoms (PCS) are common consequences in older children and adults and include somatic (eg, headache, dizziness), cognitive (eg, confusion, poor concentration), affective (eg, anxiety, sadness), and sleep (eg, fatigue, drowsiness) problems.^4,5^ In children aged 6 years and older PCS are quantified using caregiver or self-report questionnaires, emerge within hours or days after injury, and typically resolve within 10 to 14 days^6^; however approximately 31% of children have persisting symptoms 1 month after injury,^7,8^ and these are associated with adverse long-term physical, emotional, social, and academic quality of life outcomes.^9,10^ To optimize recovery, it is critical to track and treat symptoms. However, comparatively little is known about how PCS present and evolve after early childhood concussion.

To date, research on PCS in early childhood concussion has mainly relied on measures validated in school-age children,^11,12^ adaptations thereof,^13,14,15,16^ or retrospective health record review.^16,17^ Some studies show that young children experience typical PCS, such as physical (eg, headache) and sleep (eg, fatigue, drowsiness) disturbances,^13,15^ while others suggest that very young children display unique manifestations, such as stomachaches, increased dependence, and clinginess.^14,17^ Discrepancies across studies may be related to methodological limitations. For example, using adaptations of questionnaires validated in older children may overlook manifestations that are unique to young children.^18^ When caregivers are provided with specific, tangible examples of how PCS could manifest in young children, they tend to report behavioral changes (eg, excessive crying, tantrums, clinginess) that are thought to reflect underlying symptoms due to the child’s inability to verbally communicate abstract sensations and concepts (eg, dizziness, feeling foggy).^14,17^ In response to the need for a more developmentally appropriate approach, the Report of Early Childhood Traumatic Injury Observations & Symptoms (REACTIONS) inventory provides examples of observable manifestations and behaviors related to PCS. In a study of 98 participants, children aged 0 to 2 years had different patterns of PCS using the REACTIONS inventory compared with those aged 3 to 8 years.^19^ Behavioral manifestations (irritability, crying, comfort-seeking) were especially salient in the acute phase for the younger age group, while physical manifestations (headache, nausea) were prominent for the older group. However, the nature and evolution of early childhood PCS remains poorly characterized due to retrospective designs, small samples, the inclusion of school-aged children that may confound the findings, and lack of comparison groups to confirm whether symptoms are concussion-specific, general responses to traumatic injury, or expected behavioral variations typical of early childhood.

The objective of this study was to comprehensively assess PCS after early childhood concussion using a developmentally appropriate measure. Specifically, we aimed to document the amount, type, and evolution of PCS across time (before injury, acutely [in the ED], and 10 days, 1 month, and 3 months after injury) in young children (aged 6-72 months) with concussion compared with children with orthopedic injury (OI) and uninjured children from the community (control group). We hypothesized that the 3 groups would have comparable preinjury PCS. Children with early childhood concussion were expected to have, in total, more PCS than both the OI and CC groups up to and including 1 month after injury, with physical and behavioral symptoms especially prominent. We also expected the OI group to have more symptoms than the uninjured control group, but fewer than the concussion group. By 3 months after injury, we anticipated comparable PCS levels across all groups based on previous research.

## Methods

This cohort study was approved by the research ethics boards of all participating sites. Informed consent was obtained from caregivers. The study is reported in accordance with the Strengthening the Reporting of Observational Studies in Epidemiology (STROBE) reporting guideline.

### Study Design

The Kids’ Outcomes and Long-Term Abilities (KOALA) project^20^ is a prospective cohort study conducted at 4 tertiary, urban, pediatric hospitals: CHU Sainte-Justine (Quebec, Canada), Montreal Children’s Hospital (Quebec, Canada), Alberta Children’s Hospital (Alberta, Canada), and Nationwide Children’s Hospital (Ohio). This study presents analyses from data collected between December 2018 to December 2022.

### Participants and Study Protocol

Children aged 6 months to younger than 6 years (72 months) with concussion or OI were recruited when they presented to the ED within 48 hours of injury. Uninjured children from the community were recruited through advertisement in the Healthy Infants and Children Clinical Research Program (Calgary, Canada) and in 8 daycares (Montreal, Canada). Inclusion criteria for mTBI were documented, nonintentional, traumatic event resulting in a nonpenetrating injury; Glasgow Coma Scale score between 13 and 15 using an age-appropriate scale; and at least 1 of the following: palpable skull fracture, headache, or altered mental status (agitation, somnolence, slow response, repetitive questioning, excessive irritability, or loss of consciousness). The current analyses included only participants with uncomplicated mTBI, thus excluding 10 patients who had skull fractures or intracranial lesions. The inclusion criteria for the OI group were upper or lower limb trauma leading to a final diagnosis of simple fracture, sprain, contusion, or unspecified trauma to the extremity and absence of TBI. The inclusion criteria for the uninjured control group were to be within study age range and to have a typical course of development. The following exclusion criteria applied to all groups: (1) hypoxia, hypotension, or shock; (2) administration of sedative medication; (3) neurosurgical intervention; (4) injury related to suspected or known abuse or assault; (5) legal guardian not present or child in foster care; (6) insufficient parental mastery of English or French; (7) diagnosed, severe, congenital, neurologic, developmental, psychiatric, or metabolic disorder; (8) gestational age younger than 37 weeks; and (9) history of prior TBI. Patients fulfilling the inclusion and exclusion criteria were identified and invited to participate. After consent, caregivers were asked to complete the REACTIONS inventory retrospectively (preinjury), and acutely (ED, within 48 hours), postacutely (10 days: 6-14 days), 1 month (23-44 days) and 3 months (75-104 days) after injury. For the control group, the same time points were used after recruitment. Caregivers completed an in-house questionnaire to document demographics and developmental history, and injury characteristics were collected using a case report form completed by research staff and the attending physician when necessary. Demographic characteristics included self-reported family cultural origin, classified as Arab, Asian, Black, Indigenous, Latinx, and White. Cultural origin was assessed because of the importance of establishing whether the sample was representative of the broader population and the possibility that concussion outcome may vary by race and ethnicity.^21,22^

### Primary Outcome Measure

Symptoms were assessed using the REACTIONS inventory (106-item version), which assesses 17 PCS across 3 domains: cognitive (attention and concentration, memory, and processing speed), physical (headache, nausea, balance and coordination, fatigue, sleep, vision, and sensitivity to light, noise, or touch), and behavioral (irritability, mood and motivation, anxiety, regression, and comfort). Each symptom is accompanied by a list of age-appropriate, observable manifestations (eTable 1 in Supplement 1) for a total of 106 items. Caregivers completed the retrospective time point assessment by indicating whether a manifestation was present or not prior to injury or recruitment. They then complete the inventory based on changes they notice in their child since the injury or recruitment, selecting yes only if the symptom and its associated manifestations are more pronounced or frequent than before the injury or recruitment. They can also rate symptom severity (mild, moderate, severe). REACTIONS was completed in print or electronically using REDCap electronic data capture (Vanderbilt University) hosted at CHU Sainte-Justine.^23^ Details on content development and internal consistency have been previously published.^19^ To quantify PCS, REACTIONS scores were analyzed at the symptom level, where each of 17 symptoms are either endorsed or not (score of 0 or 1); at the domain level, where all affirmed constituent symptoms are tallied (ranges: cognitive, 0-3; physical, 0-9; behavioral, 0-5); and at the scale level, which is a tally of all possible symptoms across the 3 domains (range, 0-17), resulting in a total symptoms score, with higher score indicating worse symptoms.

### Statistical Analysis

Participant demographics and injury characteristics are presented descriptively using means, SDs, frequencies, and percentages across groups (concussion, OI, control). Descriptive data analyses were performed using IBM SPSS Statistics for Windows 27.0. Pearson χ^2^ test was used for categorical variables, and either 1-way analysis of variance or the Kruskal-Wallis test was used for continuous variables, based on their distributions.

PCS were summarized by group and study time points using descriptive statistics and illustrated using graphs. To investigate group differences in PCS across time, binary logistic and ordinal regression models with cluster-adjusted SE to correct for repeated measurement were performed, with PCS scores treated as ordinal categorical variables. Separate models were fitted for each symptom (n = 17), domain (n = 3), and total (scale-level) REACTIONS score, resulting in 21 different models. Main factors of interest include group (concussion, OI, control) by time (preinjury, ED, 10-day, 1-month, 3-month assessments) interactions. All models were adjusted for age and sex. Postmodel fit contrasts were then performed to detail all possible pairwise comparisons of time and group categories. Effect sizes are expressed in terms of adjusted odds ratio (ORs) and associated 95% CIs. These analyses were performed between March 2023 and January 2024, using *R* statistical software version 4.2.2 (R Project for Statistical Computing). *P* values were 2-sided, and statistical significance was set at *P* = .05.

## Results

### Child and Caregiver Characteristics and PCS Patterns

Of 961 children meeting inclusion criteria, 343 provided consent and were enrolled, and 303 children (mean [SD] age, 35.8 [20.2] months; 152 [50.2%] male) were included in this analysis. Of these, 174 had a concussion (mean [SD] age, 33.3 [19.9] months), 60 had an OI (mean [SD] age, 38.4 [19.8] months) and 69 were uninjured controls (mean [SD] age, 39.7 [20.8] months). Child, caregiver, and injury characteristics are presented in Table 1. The concussion group was significantly younger and had less parent education than the control group (Table 1). There were no differences between the concussion and OI groups for age or parent education (Table 1). Most responders were mothers (255 responders [87.9%]). The predominant concussion mechanism was non–sports-related injury (116 participants [84.7%]), especially falls from a height (54 participants [47.0%]). Domain- and scale-level PCS by group are presented in eFigure 1 in Supplement 1. Children who experienced early childhood concussion had a mean (SD) total of 8.47 (4.26) PCS acutely, 8.66 (4.64) PCS at 10 days, 6.98 (4.53) PCS at 1 month, and 5.96 (3.91) PCS at 3 months. The OI group had a mean (SD) of 4.45 (3.59) PCS acutely, 5.03 (4.17) PCS at 10 days, 4.16 (3.50) PCS at 1 month, and 3.57 (3.56) PCS at 3 months. The CC group had a mean (SD) of 3.27 (3.45) PCS acutely, 4.02 (3.94) PCS at 10 days, 3.38 (3.70) PCS at 1 month, and 3.58 (3.81) PCS at 3 months. Children with concussion showed more PCS in total and in each domain after their injury than both comparison groups. In the concussion group, total, cognitive, and behavioral PCS increased from before their injury to ED and peaked at 10 days after injury before decreasing at the 1-month and 3-month time points. Physical PCS increased from before injury, peaked in the ED, and then gradually decreased at 10 days, 1 months, and 3 months. The mean number of symptoms at the domain- and scale-level for each group are presented in Table 2.

**Table 1.** Child, Caregiver, and Injury Characteristics by Group

Variable	Injury group, No. (%)				
	Concussion (n = 174)	OI (n = 60)	Uninjured control (n = 69)	*P* value
Child sex				
Female	81 (46.6)	33 (55.0)	37 (53.6)	.41
Male	93 (53.4)	27 (45.0)	32 (46.4)	
Child age at injury, mean (SD), mo	33.3 (19.9)	38.4 (19.8)	39.7 (20.8)	.05
Caregiver relationship to child				
Mother	143 (87.7)	51 (87.9)	61 (88.4)	.95
Father	20 (12.3)	7 (12.1)	8 (11.6)	
Missing	11 (6.3)	2 (3.3)	0	
Caregiver age, mean (SD), y				
Mother	31.3 (7.3)	32.0 (5.4)	32.8 (4.2)	.31
Father	33.1 (7.9)	33.9 (6.5)	34.7 (4.9)	.35
Caregiver highest level of education				
No high school diploma	7 (5.1)	1 (2.3)	0	<.001
High school	14 (10.3)	2 (4.7)	1 (1.5)	
CEGEP or professional diploma^a^	37 (27.2)	12 (27.9)	13 (19.4)	
Undergraduate	50 (36.8)	12 (27.9)	17 (25.4)	
Graduate	28 (20.6)	16 (37.2)	36 (53.7)	
Missing	38 (21.8)	17 (28.3)	2 (2.9)	
Family cultural origins				
Arab	3 (2.3)	2 (4.7)	1 (1.5)	.04
Asian	11 (8.5)	5 (11.6)	8 (11.9)	
Black	12 (9.2)	5 (11.6)	4 (6.0)	
Indigenous	19 (14.1)	1 (2.3)	2 (3.0)	
Latinx	14 (14.6)	2 (4.7)	2 (3.0)	
White	71 (54.6)	28 (65.1)	50 (74.6)	
Missing	44 (25.3)	17 (28.3)	2 (2.9)	
Mechanism of injury^b^				
Sport or recreational play	21 (15.3)	12 (27.9)	NA	<.001
Non-sport-related injury or fall	116 (84.7)	28 (65.1)		
Other	0	3 (7.0)		
Type of sport or recreational play				
Recreational play	16 (76.2)	9 (75.0)	NA	.23
Bicycling	2 (9.5)	0		
Soccer	1 (4.8)	0		
Skating	1 (4.8)	0		
Skateboarding	1 (4.8)	0		
Trampoline	0	2 (16.7)		
Tobogganing	0	1 (8.3)		
Type of non-sport-related injury or fall^c^				
Fall from height	54 (47.0)	4 (17.4)	NA	.006
Slipped/fell/tripped on floor/ground	25 (21.7)	14 (60.9)		
Struck against household object	13 (11.3)	1 (4.3)		
Struck by object	12 (10.4)	3 (13.0)		
Fall down stairs	6 (5.2)	1 (4.3)		
Struck head against wall/door	5 (4.3)	0		
Injury involved a fall	125 (91.2)	23 (56.1)	NA	<.001
Glasgow Coma Scale score				
14	3 (1.7)	0	NA	.34
15	173 (98.3)	60 (100)		
Loss of consciousness				
No	138 (79.3)	60 (100)	NA	<.001
Yes	30 (17.2)	0		
Unknown	6 (3.4)	0		

Abbreviations: CEGEP, College of General and Professional Teaching; NA, not applicable; OI, orthopedic injury.

^a^ CEGEP is part of the Quebec education system. Students attend CEGEP after high school grade 11 for 2 or 3 years, either to complete a diploma leading to the job market or in preparation for university.

^b^ Data were available for 180 participants.

^c^ Data were available for 138 participants.

**Table 2.** Mean Number of Symptoms by Domain and Group at Each Time Point

Time point	Symptoms, mean (SD)											
	Total			Cognitive			Physical			Behavioral		
	Concussion	OI	Control	Concussion	OI	Control	Concussion	OI	Control	Concussion	OI	Control
Preinjury	5.81 (3.93)	4.40 (3.86)	4.37 (3.89)	1.19 (1.00)	0.85 (1.01)	1.20 (0.96)	2.35 (2.27)	1.86 (2.21)	1.42 (1.69)	2.31 (1.50)	1.89 (1.40)	1.95 (1.60)
ED	8.47 (4.26)	4.45 (3.59)	3.27 (3.45)	1.42 (1.15)	0.67 (0.87)	0.94 (1.00)	4.26 (2.34)	1.52 (1.87)	0.97 (1.46)	2.87 (1.57)	2.19 (1.67)	1.51 (1.41)
10 d	8.66 (4.64)	5.03 (4.17)	4.02 (3.94)	1.62 (1.10)	0.86 (1.03)	1.06 (1.06)	4.17 (2.77)	1.71 (1.97)	1.29 (1.71)	3.01 (1.49)	2.36 (1.74)	1.76 (1.70)
1 mo	6.98 (4.53)	4.16 (3.50)	3.38 (3.70)	1.03 (1.06)	0.71 (0.96)	0.68 (0.91)	3.35 (2.56)	1.56 (1.79)	1.09 (1.63)	2.64 (1.55)	1.89 (1.48)	1.65 (1.71)
3 mo	5.96 (3.91)	3.57 (3.56)	3.58 (3.81)	1.05 (1.10)	0.72 (1.08)	0.78 (0.98)	2.79 (2.42)	1.29 (1.61)	1.20 (1.88)	2.20 (1.73)	1.58 (1.52)	1.67 (1.51)

Abbreviation: OI, orthopedic injury.

For children with concussion, attention and concentration consistently ranked as the most frequently endorsed cognitive symptom at all postinjury time points (ED, 68.6%; 10 days, 76.2%; 1 month, 61.5%; 3 months, 53.3%). In the ED, headache (73.6%) and fatigue and drowsiness (67.9%) were the most frequently endorsed physical symptoms, while sleep disturbances (10 days, 65.3%; 1 month, 63.6%; 3 months, 57.6%) and headache (10 days, 54.5%; 1 month, 40.5%; 3 months, 33.9%) were frequent at subsequent time points. In the ED and at 10 days, the 2 most common behavioral symptoms were irritability (ED, 74.6%; 10 days, 81.3%) and comfort-seeking (ED, 69.7%; 10 days, 70.7%). At subsequent time points, the 2 most frequently endorsed behavioral symptoms were irritability (1 month, 74.0%; 3 months, 57.6%) and anxiety (1 month, 60.3%; 3 months, 57.6%). Across the 17 symptoms, irritability was consistently the most frequently reported symptom at ED, 10-day, and 1-month assessments. At 3 months, sleep disturbances, irritability, and anxiety were equally prevalent, sharing the top position for frequency of endorsement. The symptom-level patterns for the groups are presented in eFigure 2 in Supplement 1, and the percentages of children with each individual symptom are in eTable 2 in Supplement 1.

### Group Differences in PCS Over Time

#### Scale- and Domain-Level Differences Between Groups

Group-by-time interaction and group differences were significant in regression models for all scale- and domain-level scores (cognitive, physical, and behavioral). Further examination of postmodel fit contrasts showed no meaningful differences between the concussion group and either comparison group at the preinjury time point (OR vs OI, 1.73 [95% CI, 1.01-2.96]; OR vs control, 1.48 [95% CI, 0.86-2.57]). Differences between the concussion group and the OI and control groups were most evident acutely (OR vs OI, 4.33 [95% CI, 2.44-7.69]; OR vs control, 7.28 [95% CI, 3.80-13.93]) and at 10 days (OR vs OI, 4.44 [95% CI, 2.17-9.06]; OR vs control, 5.94 [95% CI, 3.22-10.94]), whereas differences were smaller but nonetheless significant at the 1-month (OR vs OI, 2.70 [95% CI, 1.56-4.68]; OR vs control, 4.32 [95% CI, 2.36-7.92]) and 3-month (OR vs OI, 2.61 [95% CI, 1.30-5.25]; OR vs control, 2.40 [95% CI, 1.36-4.24]) time points. In the physical domain, the most substantial differences were found acutely compared with both comparison groups (OR vs OI, 6.80 [95% CI, 3.58-12.90]; OR vs control, 11.49 [95% CI, 6.34-20.84]). Physical domain group differences remained significant at 10 days (OR vs OI, 5.90 [95% CI, 2.94-11.84]; OR vs control, 7.42 [95% CI, 3.99-13.81]), 1 month (OR vs OI, 3.43 [95% CI, 1.94-6.06]; OR vs control, 6.15 [95% CI, 3.36-11.27]), and 3 months (OR vs OI, 3.31 [95% CI, 1.77-6.20]; OR vs control, 3.89 [95% CI, 2.16-7.01]). In the cognitive domain, significant group differences were found acutely (OR vs OI, 3.55 [95% CI, 1.73-7.31]; OR vs control, 2.30 [95% CI, 1.05-5.01]) and at 10 days (OR vs OI, 3.76 [95% CI, 1.63-8.64]; OR vs control, 2.42 [95% CI, 1.29-4.54]). In the behavioral domain, significant group differences compared with the control group were observed at the ED (OR, 3.46 [95% CI, 1.86-6.43]), 10-day (OR, 3.63 [95% CI, 2.04-6.44]), and 1-month (OR, 2.94 [95% CI, 1.60-5.42]) time points. Compared with the OI group, significant differences emerged at 10 days (OR, 2.20 [95% CI, 1.10-4.43]) and 1 month (OR, 2.14 [95% CI, 1.25-3.66]). Full details of the scale- and domain-level group comparisons can be found in the Figure and Table 3.

**Figure.**
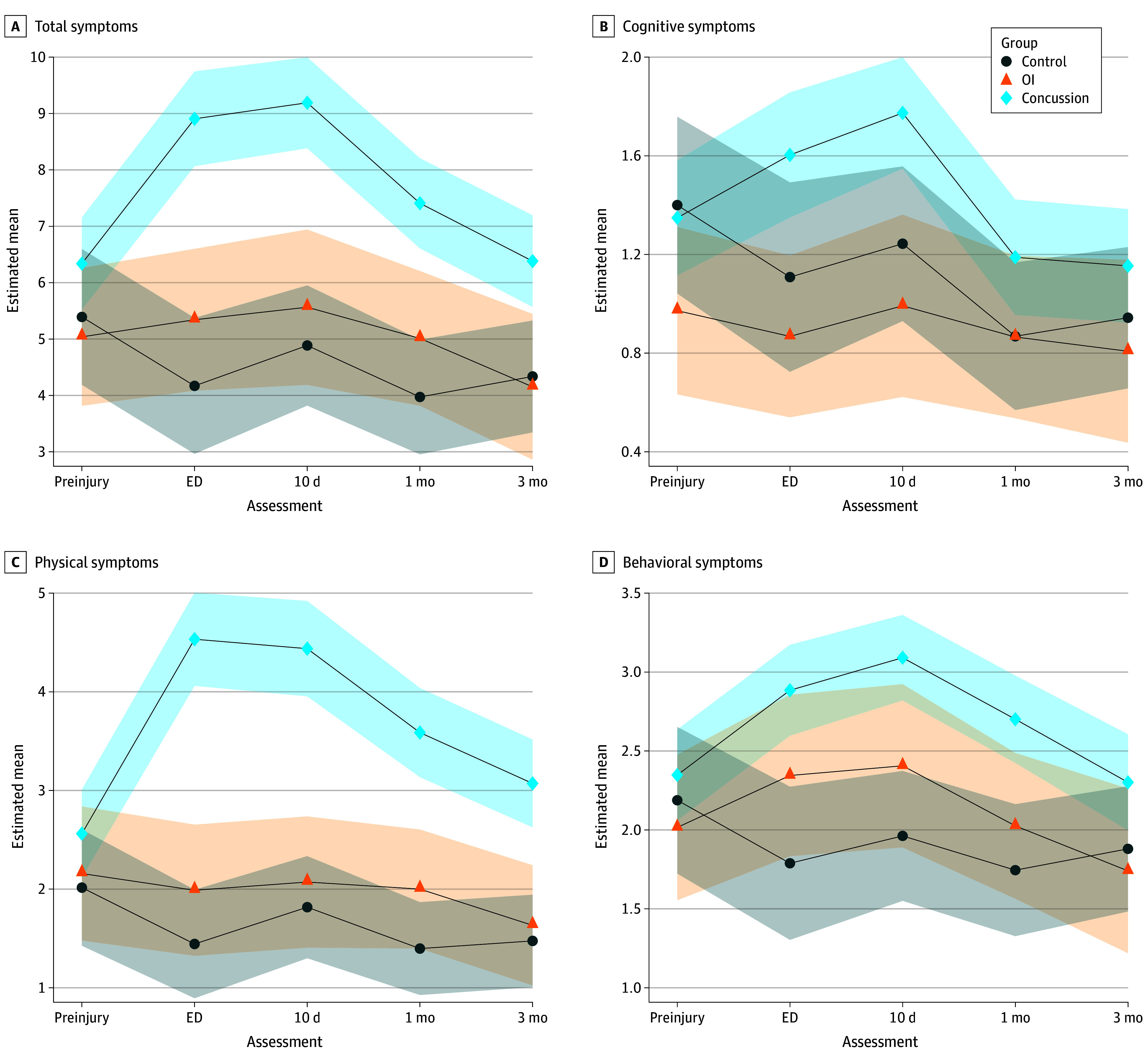
Trends for Scale- and Domain-Level Postconcussive Symptoms (PCS) by Injury Group Estimated trajectories from model fits (estimated mean of PCS over time) at the scale (total symptoms) and domain-level (cognitive, physical, behavioral symptoms) by group. ED indicates emergency department; OI, orthopedic injury. Adjusted to age (34 months) and female sex.

**Table 3.** Domain- and Scale-Level Postmodel Fit Contrasts Between Concussion Group and Comparison Groups per Time Point

Domain	Preinjury		ED		10 d		1 mo		3 mo	
	OR (95% CI)	*P* value	OR (95% CI)	*P* value	OR (95% CI)	*P* value	OR (95% CI)	*P* value	OR (95% CI)	*P* value
**Concussion vs OI**										
Cognitive	1.92 (0.98-3.76)	.06	3.55 (1.73-7.31)	<.001	3.76 (1.63-8.64)	.002	1.79 (0.88-3.63)	.11	1.88 (0.76-4.65)	.17
Physical	1.39 (0.71-2.74)	.34	6.80 (3.58-12.90)	<.001	5.90 (2.94-11.84)	<.001	3.43 (1.94-6.06)	<.001	3.31 (1.77-6.20)	<.001
Behavioral	1.45 (0.87-2.42)	.16	1.85 (0.94-3.62)	.07	2.20 (1.10-4.43)	.03	2.14 (1.25-3.66)	.006	1.88 (0.92-3.84)	.08
Total	1.73 (1.01-2.96)	.048	4.33 (2.44-7.69)	<.001	4.44 (2.17-9.06)	<.001	2.70 (1.56-4.68)	<.001	2.61 (1.30-5.25)	.007
**Concussion vs uninjured control**										
Cognitive	0.92 (0.51-1.66)	.78	2.30 (1.05-5.01)	.04	2.42 (1.29-4.54)	.006	1.78 (0.92-3.43)	.09	1.45 (0.75-2.82)	.27
Physical	1.58 (0.90-2.75)	.11	11.49 (6.34-20.84)	<.001	7.42 (3.99-13.81)	<.001	6.15 (3.36-11.27)	<.001	3.89 (2.16-7.01)	<.001
Behavioral	1.20 (0.68-2.10)	.54	3.46 (1.86-6.43)	<.001	3.63 (2.04-6.44)	<.001	2.94 (1.60-5.42)	<.001	1.61 (0.91-2.84)	.10
Total	1.48 (0.86-2.57)	.16	7.28 (3.80-13.93)	<.001	5.94 (3.22-10.94)	<.001	4.32 (2.36-7.92)	<.001	2.40 (1.36-4.24)	.003

Abbreviations: OI, orthopedic injury; OR, odds ratio.

#### Symptom-Level Differences Between Groups

Group-by-time interactions were significant in statistical models for 10 of the 17 REACTIONS symptoms (eFigure 3 in Supplement 1). Group differences were significant in statistical models for 12 of 17 symptoms. Further examination of postmodel fit contrasts revealed that group differences (concussion vs OI, control, or both) were found acutely in the ED (significant for 11 symptoms: attention and concentration, processing speed, headache, nausea, balance and coordination, fatigue and drowsiness, sleep, vision, sensitivity to light, irritability, and mood and motivation), at 10 days (significant for 12 symptoms: attention and concentration, processing speed, headache, nausea, balance and coordination, fatigue and drowsiness, sleep, vision, sensitivity to light and noise, irritability, and mood and motivation), and at 1 month (significant for 11 symptoms: attention and concentration, headache, nausea, balance, fatigue and drowsiness, sleep, vision, sensitivity to light and noise, irritability, and mood and motivation). Group differences continued to be significant later in recovery at 3 months (significant for 7 symptoms: headache, nausea, balance, fatigue and drowsiness, sleep, vision, sensitivity to noise).

Across time points and compared with both comparison groups, the concussion group had significantly greater odds for headache (OR vs OI, 13.61 [95% CI, 5.48-33.85]; OR vs control, 26.13 [8.77-77.89]), nausea (OR vs OI, 12.19 [95% CI, 4.52-32.92]; OR vs control, 67.76 [95% CI, 9.10-504.65]), and fatigue and drowsiness (OR vs OI, 5.86 [95% CI, 2.68-12.84]; OR vs control, 15.30 [95% CI, 5.63-41.61]) at the acute ED time point. At 10 days, the concussion group had significantly higher odds of headache (OR vs OI, 22.69 [95% CI, 5.20-98.99]; OR vs control, 6.72 [95% CI, 3.07-14.67]), fatigue and drowsiness (OR vs OI, 3.75 [95% CI, 1.65-8.54]; OR vs control, 4.63 [95% CI, 2.25-9.52]), and vision symptoms (OR vs OI, 5.06 [95% CI, 1.86-13.77]; OR vs control, 6.73 [95% CI, 2.75-16.47]). At the ED, 10-day, and 1-month time points, compared with the control group only, substantially greater odds were found for sleep (ED: OR, 5.29 [95% CI, 2.33-12.05]; 10 days: OR, 3.13 [95% CI, 1.65-5.95]; 1 month: OR, 3.70 [95% CI, 1.91-7.15]), irritability (ED: OR, 4.45 [95% CI, 2.13-9.29]; 10 days, 6.85 [95% CI, 3.46-13.55]; 1 month: OR, 3.76 [95% CI, 1.98-7.14]), and mood and motivation (ED: OR, 7.12 [95% CI, 2.64-19.22]; 10 days: OR, 3.43 [95% CI, 1.68-6.98]; 1 month: OR, 3.64 [95% CI, 1.70-7.77]). Full details of all postmodel fit contrasts are presented in eTable 3 in Supplement 1.

## Discussion

This cohort study found that PCS significantly increased after concussion compared with preinjury ratings and were significantly elevated compared with children with OI and uninjured children from the community. As hypothesized, children with early childhood concussion had more PCS acutely and at 10 days and 1 month after injury. Counter to expectations, physical PCS remained significantly elevated in the concussion group even after 3 months from their injury, and included headache, nausea, balance difficulties, fatigue and drowsiness, sleep disturbances, vision difficulties, and sensitivity to noise. Group differences were particularly large acutely and at 10 days after injury, and although symptoms diminished with time, children with concussion were still more likely to exhibit PCS at 1 and 3 months after their injury than either comparison group. The pattern of PCS evolution differed across domains. Cognitive and behavioral symptoms increased from preinjury levels, peaking at 10 days after injury and then gradually declining at 1 month, with further reductions evident at 3 months, as would be expected over the course of concussion recovery. Physical symptoms were highest at the ED and then progressively diminished over time. Overt physical symptoms, such as nausea, are probably easier to observe in the acute period. Conversely, cognitive (eg, attention and concentration) and behavioral symptoms (eg, irritability, comfort-seeking) may only become apparent to caregivers over time and in the day-to-day environment. Although PCS trends for school-aged children have been well established,^7,24^ studies of concussion in young children have mainly focused on symptoms immediately after the injury.^11,13,25^ The findings from this cohort study confirm the presence of PCS after early concussion and track their evolution, indicating a clear symptom burden compared with children with no brain injury.

Children with concussion displayed more inattention, slowing down, headache, nausea, imbalance, fatigue and drowsiness, poor sleep, vision problems, sensitivity to light and noise, and irritability compared with children with OI and uninjured children from the community. These are all typical, well-documented symptoms of concussion in school-aged children^4,26^ and have also been reported in some studies of early childhood concusion.^13,14,15,17^ Our findings suggest that using an observational PCS inventory that provides caregivers with guidance as to what manifestations and behaviors can be expected was helpful in identifying symptoms in their young child after a concussion. It is noteworthy that irritability emerged as a prominent symptom, probably serving as one of the primary means through which young children communicate feelings of being unwell and representing a range of possible underlying symptoms. Several behavioral manifestations of comfort-seeking (eg, wants to be held, gets upset if separated from parent) and poor mood and motivation (eg, cries a lot, is withdrawn and isolated), which are not included in other PCS questionnaires, were also reported. These findings strengthen the evidence from smaller or retrospective studies showing that early childhood concussion is associated with behavioral manifestations unique to this developmental period, including more fussiness, wanting to be cuddled more, increased crying, and poor feeding.^11,17,18,19^ Given their limited verbal and cognitive abilities, behavioral manifestations, such as regression, comfort-seeking, or excessive crying, may be the only ways a young child can convey that they feel unwell after injury. Documenting these behavioral manifestations through direct observation could be the key to tracking PCS and facilitating concussion diagnosis in young children.

As hypothesized, children with OI also endorsed several symptoms, with their levels of PCS falling between those of children with concussion and uninjured children. Numerous studies have documented sleep, anxiety, and irritability in children with OI,^27,28,29,30,31^ and they are recognized as constituting an optimal comparison group for concussion due to their shared experience of traumatic injury, pain, and stress.^32^ These commonalities are reinforced in our study, given their comparable number of acute behavioral symptoms. However, children with concussion nevertheless exhibited more PCS than children with OI, even 3 months after the injury, when headache, balance problems, and fatigue continued to be observed, suggesting persisting physical symptoms beyond the expected 1-month recovery period after concussion.^7^

There is debate as to whether young children exhibit PCS to the same extent as older children. Previous studies have been equivocal, with some showing that young children experience fewer PCS than school-aged children (2-5 vs 8 symptoms)^11,13,33^ and others suggesting comparable levels.^14^ In this study, children who experienced early childhood concussion had a mean of 8 PCS acutely and at 10 days, 6 symptoms at 1 month, and 5 symptoms at 3 months. While not directly comparable with ratings in older children because of measurement differences and the specific focus of this study, the findings clearly indicate that children with early childhood concussion have an important symptom burden, and that the rate of PCS is significantly higher than in children with OI or than would be expected as part of typical development in uninjured children (between 3 and 5 symptoms).

To our knowledge, this is the first study to prospectively map the trajectory of PCS over a 3-month postinjury period using a developmentally appropriate measure in early childhood. The conclusion that children with early childhood concussion have notable PCS following their injury is strengthened by the study design, including preinjury ratings and 2 comparison groups.

### Limitations

This study has some limitations. First, the concussion and the community comparison groups differed significantly in terms of child age and caregiver education. Although age was controlled in the analyses to attenuate this difference, caregivers’ perceptions of PCS may vary with age. However, given the observational nature of REACTIONS, caregivers could select manifestations suitable to their child’s age and thus the age difference is unlikely to significantly affect the accuracy of their PCS reporting. Second, uninjured participants were only recruited in Canada, potentially limiting generalization to controls from the US. However, given that all cities involved were large urban areas, we anticipate minimal impact on overall generalizability. Third, as with all studies reporting preinjury PCS retrospectively, the possibility of caregiver recall bias cannot be excluded. Fourth, the current findings may not apply to all cases; children seen in the ED may differ from those who consult their family physician or do not seek medical care. However, 98% of children with concussion had a GCS of 15, suggesting they are unlikely to represent a more severely injured group. Fifth, some missing data were present in terms of injury characteristics. Sixth, the sample is fairly homogeneous and characterized by predominantly White families of higher socioeconomic status. Family cultural origins were significantly different between groups and future studies should aim to enhance sample diversity.

## Conclusions

In this early childhood cohort study, PCS were significantly elevated following concussion compared with preinjury ratings and compared with children with orthopedic injuries and uninjured community peers up to 3 months after injury. This study enhances our understanding of PCS in infants, toddlers, and preschool children, reinforcing the idea that early childhood concussion is not benign. The observed symptoms cannot be attributed to general injury or typical developmental factors. Future research should investigate risk factors and modifiers of early childhood concussion outcome using developmentally appropriate approaches.

## Notes

Supplement 1. **eTable 1.** REACTIONS Symptoms and Examples of Manifestations **eTable 2.** Percentage of Children With PCS at Each Time Point by Group **eTable 3.** Symptom-Level Post Model Fit Contrasts Between Concussion Comparison Groups per Time Point **eFigure 1.** Distribution of PCS at the Scale- and Domain-Level by Group and Time **eFigure 2.** Percentage of Caregivers Endorsing Different Symptoms by Group **eFigure 3.** Symptom-Level Differences Between Groups Based on Wald Test From Regression Model Fit

Supplement 2.
PERC KOALA Study Members

Supplement 3.
Data Sharing Statement
