# Supplementary material for: Postconcussive Symptoms After Early Childhood Concussion
Source: JAMA Netw Open. 2024 Mar 21;7(3):e243182. doi: 10.1001/jamanetworkopen.2024.3182 (PMC10958232; doi:10.1001/jamanetworkopen.2024.3182)
Supplement: Supplement 1. — eTable 1. REACTIONS Symptoms and Examples of Manifestations eTable 2. Percentage of Children With PCS at Each Time Point by Group eTable 3. Symptom-Level Post Model Fit Contrasts Between Concussion Comparison Groups per Time Point eFigure 1. Distribution of PCS at the Scale- and Domain-Level by Group and Time eFigure 2. Percentage of Caregivers Endorsing Different Symptoms by Group eFigure 3. Symptom-Level Differences Between Groups Based on Wald Test From Regression Model Fit [file jamanetwopen-e243182-s001.pdf]

Supplemental Online Content

Dupont D, Tang K, Beaudoin C, et al; PERC KOALA Study. Postconcussive symptoms after early childhood concussion. *JAMA Netw Open*. 2024;7(3):e243182. doi:10.1001/jamanetworkopen.2024.3182

eTable 1. REACTIONS Symptoms and Examples of Manifestations
eTable 2. Percentage of Children With PCS at Each Time Point by Group
eTable 3. Symptom-Level Post Model Fit Contrasts Between Concussion Comparison Groups per Time Point
eFigure 1. Distribution of PCS at the Scale- and Domain-Level by Group and Time
eFigure 2. Percentage of Caregivers Endorsing Different Symptoms by Group
eFigure 3. Symptom-Level Differences Between Groups Based on Wald Test From Regression Model Fit

This supplemental material has been provided by the authors to give readers additional information about their work.

© 2024 Dupont D et al. *JAMA Network Open.*

eTable 1. REACTIONS Symptoms and Examples of Manifestations

Domain	Symptom	Example of provided manifestation
Cognitive	Attention and concentration	Needs to be reminded several times before doing what is asked.
	Memory	Can not remember or has trouble remembering a previously learnt word (e.g., word on "tip of the tongue").
	Processing speed	Repeats the same questions, requests, or sentences.
Physical	Headache	Rubs head.
	Nausea	Eats less than usual or has no appetite.
	Balance and Coordination	Is more clumsy than usual.
	Fatigue and Drowsiness	Has less energy for activities.
	Sleep	Sleeps longer at night.
	Vision	Has a strange gaze or does not fixate.
	Sensitivity to light	Does not want to go outside.
	Sensitivity to noise	Moves away from loud noises or noisy situations.
	Tactile sensitivity	Does not tolerate specific fabrics or clothing (e.g., buttons, zippers, etc.).
Behavioral	Irritability	Is irritable or grumpy.
	Mood and motivation	Expressions of emotions are amplified (e.g., feelings of joy or anger seem extreme).
	Anxiety	Stays close to parents or caregivers.
	Regression	Refuses to speak.
	Comfort	Wants to be held.

© 2024 Dupont D et al. *JAMA Network Open.*

eTable 2. Percentage of Children with PCS at Each Timepoint by Group

		Concussion					OI					CC				
**Domain**	**PCS**	**Pre**	**ED**	**10D**	**1M**	**3M**	**Pre**	**ED**	**10D**	**1M**	**3M**	**Pre**	**ED**	**10D**	**1M**	**3M**
Cognitive	Attention/ concentration	68.3	68.6	76.2	61.5	53.3	53.5	45.2	47.4	43.2	37.1	61.7	51.2	60.3	39.3	43.8
	Memory	25.6	33.7	44.7	28.9	33.3	18.4	10.8	25.7	15.8	22.6	21.6	15.2	21.6	14.6	20.0
	Processing Speed	29.9	50.0	39.1	21.6	18.8	19.0	23.8	13.5	15.0	17.1	31.0	24.3	23.2	13.2	10.2
Physical	Headache	21.1	73.6	54.5	40.5	33.9	11.4	16.3	5.0	13.6	5.6	14.3	9.3	14.5	13.1	12.5
	Nausea	18.8	62.6	47.6	36.8	26.6	6.8	11.6	25.0	15.9	11.1	8.2	2.3	11.1	6.6	9.4
	Balance	27.7	41.7	45.4	36.9	32.5	19.5	27.5	17.9	18.6	5.6	8.3	7.1	9.8	8.2	6.2
	Fatigue/Drowsiness	23.3	67.9	52.4	37.9	26.6	11.6	26.2	22.5	20.5	5.6	14.6	11.9	19.0	11.3	15.6
	Sleep	40.6	60.4	65.3	63.6	57.6	33.3	32.4	52.5	45.5	38.9	34.7	20.9	36.5	30.6	29.7
	Vision	32.5	43.6	43.1	40.0	32.5	23.8	17.5	12.8	15.9	2.8	10.6	9.8	9.7	12.9	12.5
	Sensitivity to light	10.3	24.8	22.8	17.2	9.8	2.4	4.9	13.2	4.7	8.6	4.3	2.4	3.2	1.6	6.5
	Sensitivity to noise	38.6	39.8	51.2	40.5	35.0	35.7	24.4	12.5	20.5	25.0	37.5	31.0	32.3	21.0	18.8
	Tactile sensitivity	27.6	18.9	27.9	20.8	22.0	29.3	17.5	10.0	4.5	16.7	17.0	9.8	15.9	9.7	15.6
Behavioral	Irritability	52.3	74.6	81.3	74.0	57.6	44.2	61.4	55.0	50.0	47.2	45.8	38.1	38.1	41.9	48.4
	Mood and Motivation	20.8	49.2	47.5	41.7	32.0	6.8	35.7	32.5	13.6	22.2	12.2	11.6	20.6	16.1	21.9
	Anxiety	60.9	59.0	69.4	60.3	57.6	48.8	45.0	50.0	45.5	47.2	54.2	42.9	50.8	43.5	39.1
	Regression	36.7	44.1	32.3	30.0	20.9	35.3	35.3	32.4	23.1	12.5	39.5	30.6	25.5	21.4	18.6
	Comfort	65.9	69.7	70.7	58.8	53.7	58.1	59.5	60.0	55.8	35.3	53.1	41.9	50.8	41.9	42.2

Note: Values in the table represent percentage of respondents (caregivers) reporting the specific PCS observed in their child.

© 2024 Dupont D et al. *JAMA Network Open.*

eTable 3. Symptom-Level Post Model Fit Contrasts Between Concussion Comparison Groups per Timepoint

Model	Outcome	Contrast	Timepoint	OR (95% CI)
1	Attention/Concentration	Concussion vs OI	Pre-Injury	1.76 (0.85, 3.62)
1	Attention/Concentration	Concussion vs CC	Pre-Injury	1.23 (0.61, 2.47)
1	Attention/Concentration	Concussion vs OI	ED visit	2.49 (1.21, 5.12)
1	Attention/Concentration	Concussion vs CC	ED visit	1.93 (0.94, 3.98)
1	Attention/Concentration	Concussion vs OI	10-day timepoint	3.44 (1.58, 7.51)
1	Attention/Concentration	Concussion vs CC	10-day timepoint	2.02 (1.05, 3.90)
1	Attention/Concentration	Concussion vs OI	1-month timepoint	2.07 (1.04, 4.13)
1	Attention/Concentration	Concussion vs CC	1-month timepoint	2.35 (1.24, 4.43)
1	Attention/Concentration	Concussion vs OI	3-month timepoint	1.87 (0.86, 4.07)
1	Attention/Concentration	Concussion vs CC	3-month timepoint	1.39 (0.75, 2.58)
3	Processing speed	Concussion vs OI	Pre-Injury	1.71 (0.70, 4.15)
3	Processing speed	Concussion vs CC	Pre-Injury	0.85 (0.38, 1.86)
3	Processing speed	Concussion vs OI	ED visit	3.05 (1.36, 6.86)
3	Processing speed	Concussion vs CC	ED visit	2.81 (1.19, 6.63)
3	Processing speed	Concussion vs OI	10-day timepoint	3.95 (1.42, 10.98)
3	Processing speed	Concussion vs CC	10-day timepoint	1.97 (0.95, 4.11)
3	Processing speed	Concussion vs OI	1-month timepoint	1.54 (0.58, 4.09)
3	Processing speed	Concussion vs CC	1-month timepoint	1.67 (0.66, 4.20)
3	Processing speed	Concussion vs OI	3-month timepoint	1.10 (0.40, 3.01)
3	Processing speed	Concussion vs CC	3-month timepoint	1.89 (0.71, 5.00)
4	Headache	Concussion vs OI	Pre-Injury	1.89 (0.67, 5.32)
4	Headache	Concussion vs CC	Pre-Injury	1.45 (0.59, 3.54)
4	Headache	Concussion vs OI	ED visit	13.61 (5.48, 33.85)
4	Headache	Concussion vs CC	ED visit	26.13 (8.77, 77.89)
4	Headache	Concussion vs OI	10-day timepoint	22.69 (5.20, 98.99)

© 2024 Dupont D et al. *JAMA Network Open.*

Model	Outcome	Contrast	Timepoint	OR (95% CI)
4	Headache	Concussion vs CC	10-day timepoint	6.72 (3.07, 14.67)
4	Headache	Concussion vs OI	1-month timepoint	4.21 (1.65, 10.75)
4	Headache	Concussion vs CC	1-month timepoint	4.16 (1.84, 9.38)
4	Headache	Concussion vs OI	3-month timepoint	8.67 (2.00, 37.63)
4	Headache	Concussion vs CC	3-month timepoint	3.33 (1.46, 7.59)
5	Nausea	Concussion vs OI	Pre-Injury	3.01 (0.86, 10.53)
5	Nausea	Concussion vs CC	Pre-Injury	2.48 (0.82, 7.47)
5	Nausea	Concussion vs OI	ED visit	12.19 (4.52, 32.92)
5	Nausea	Concussion vs CC	ED visit	67.76 (9.10, 504.65)
5	Nausea	Concussion vs OI	10-day timepoint	2.68 (1.20, 5.99)
5	Nausea	Concussion vs CC	10-day timepoint	7.06 (2.99, 16.65)
5	Nausea	Concussion vs OI	1-month timepoint	3.04 (1.25, 7.38)
5	Nausea	Concussion vs CC	1-month timepoint	7.99 (2.72, 23.43)
5	Nausea	Concussion vs OI	3-month timepoint	2.88 (0.94, 8.79)
5	Nausea	Concussion vs CC	3-month timepoint	3.37 (1.32, 8.55)
6	Balance	Concussion vs OI	Pre-Injury	1.43 (0.58, 3.49)
6	Balance	Concussion vs CC	Pre-Injury	3.78 (1.26, 11.39)
6	Balance	Concussion vs OI	ED visit	1.73 (0.77, 3.88)
6	Balance	Concussion vs CC	ED visit	8.65 (2.50, 29.97)
6	Balance	Concussion vs OI	10-day timepoint	3.76 (1.52, 9.30)
6	Balance	Concussion vs CC	10-day timepoint	7.25 (2.87, 18.34)
6	Balance	Concussion vs OI	1-month timepoint	2.50 (1.05, 5.95)
6	Balance	Concussion vs CC	1-month timepoint	6.06 (2.31, 15.92)
6	Balance	Concussion vs OI	3-month timepoint	8.19 (1.88, 35.63)
6	Balance	Concussion vs CC	3 month timepoint	6.70 (2.28, 19.70)
7	Fatigue and Drowiness	Concussion vs OI	Pre-Injury	2.26 (0.82, 6.24)
7	Fatigue and Drowiness	Concussion vs CC	Pre-Injury	1.72 (0.70, 4.22)

© 2024 Dupont D et al. *JAMA Network Open.*

Model	Outcome	Contrast	Timepoint	OR (95% CI)
7	Fatigue and Drowiness	Concussion vs OI	ED visit	5.86 (2.68, 12.84)
7	Fatigue and Drowiness	Concussion vs CC	ED visit	15.30 (5.63, 41.61)
7	Fatigue and Drowiness	Concussion vs OI	10-day timepoint	3.75 (1.65, 8.54)
7	Fatigue and Drowiness	Concussion vs CC	10-day timepoint	4.63 (2.25, 9.52)
7	Fatigue and Drowiness	Concussion vs OI	1-month timepoint	2.36 (1.05, 5.30)
7	Fatigue and Drowiness	Concussion vs CC	1-month timepoint	4.72 (1.99, 11.18)
7	Fatigue and Drowiness	Concussion vs OI	3-month timepoint	6.11 (1.39, 26.74)
7	Fatigue and Drowiness	Concussion vs CC	3-month timepoint	1.93 (0.88, 4.22)
8	Sleep	Concussion vs OI	Pre-Injury	1.21 (0.54, 2.69)
8	Sleep	Concussion vs CC	Pre-Injury	1.15 (0.56, 2.33)
8	Sleep	Concussion vs OI	ED visit	2.82 (1.28, 6.22)
8	Sleep	Concussion vs CC	ED visit	5.29 (2.33, 12.05)
8	Sleep	Concussion vs OI	10-day timepoint	1.65 (0.80, 3.41)
8	Sleep	Concussion vs CC	10-day timepoint	3.13 (1.65, 5.95)
8	Sleep	Concussion vs OI	1-month timepoint	2.04 (1.02, 4.08)
8	Sleep	Concussion vs CC	1-month timepoint	3.70 (1.91, 7.15)
8	Sleep	Concussion vs OI	3-month timepoint	2.12 (0.99, 4.55)
8	Sleep	Concussion vs CC	3-month timepoint	3.04 (1.58, 5.82)
9	Vision	Concussion vs OI	Pre-Injury	1.31 (0.57, 3.01)
9	Vision	Concussion vs CC	Pre-Injury	3.54 (1.30, 9.67)
9	Vision	Concussion vs OI	ED visit	3.24 (1.32, 7.99)
9	Vision	Concussion vs CC	ED visit	6.42 (2.10, 19.63)
9	Vision	Concussion vs OI	10-day timepoint	5.06 (1.86, 13.77)
9	Vision	Concussion vs CC	10-day timepoint	6.73 (2.75, 16.47)
9	Vision	Concussion vs OI	1-month timepoint	3.45 (1.41, 8.44)
9	Vision	Concussion vs CC	1-month timepoint	4.15 (1.81, 9.51)
9	Vision	Concussion vs OI	3-month timepoint	17.17 (2.20, 133.78)

© 2024 Dupont D et al. *JAMA Network Open.*

Model	Outcome	Contrast	Timepoint	OR (95% CI)
9	Vision	Concussion vs CC	3-month timepoint	3.11 (1.32, 7.31)
10	Sensitivity to light	Concussion vs OI	Pre-Injury	4.58 (0.57, 36.45)
10	Sensitivity to light	Concussion vs CC	Pre-Injury	2.54 (0.54, 12.00)
10	Sensitivity to light	Concussion vs OI	ED visit	6.27 (1.42, 27.74)
10	Sensitivity to light	Concussion vs CC	ED visit	12.99 (1.66, 101.78)
10	Sensitivity to light	Concussion vs OI	10-day timepoint	1.92 (0.69, 5.38)
10	Sensitivity to light	Concussion vs CC	10-day timepoint	8.70 (1.99, 38.02)
10	Sensitivity to light	Concussion vs OI	1-month timepoint	4.21 (0.95, 18.63)
10	Sensitivity to light	Concussion vs CC	1-month timepoint	12.19 (1.60, 92.92)
10	Sensitivity to light	Concussion vs OI	3-month timepoint	1.15 (0.31, 4.31)
10	Sensitivity to light	Concussion vs CC	3-month timepoint	1.53 (0.47, 5.03)
11	Sensitivity to noise	Concussion vs OI	Pre-Injury	1.02 (0.48, 2.16)
11	Sensitivity to noise	Concussion vs CC	Pre-Injury	0.92 (0.46, 1.85)
11	Sensitivity to noise	Concussion vs OI	ED visit	1.88 (0.84, 4.20)
11	Sensitivity to noise	Concussion vs CC	ED visit	1.31 (0.61, 2.80)
11	Sensitivity to noise	Concussion vs OI	10-day timepoint	7.23 (2.69, 19.45)
11	Sensitivity to noise	Concussion vs CC	10-day timepoint	2.06 (1.07, 3.97)
11	Sensitivity to noise	Concussion vs OI	1-month timepoint	2.56 (1.15, 5.74)
11	Sensitivity to noise	Concussion vs CC	1-month timepoint	2.36 (1.15, 4.84)
11	Sensitivity to noise	Concussion vs OI	3-month timepoint	1.58 (0.67, 3.73)
11	Sensitivity to noise	Concussion vs CC	3-month timepoint	2.17 (1.04, 4.54)
13	Irritability	Concussion vs OI	Pre-Injury	1.30 (0.65, 2.63)
13	Irritability	Concussion vs CC	Pre-Injury	1.20 (0.62, 2.32)
13	Irritability	Concussion vs OI	ED visit	1.74 (0.84, 3.57)
13	Irritability	Concussion vs CC	ED visit	4.45 (2.13, 9.29)
13	Irritability	Concussion vs OI	10-day timepoint	3.47 (1.59, 7.58)
13	Irritability	Concussion vs CC	10-day timepoint	6.85 (3.46, 13.55)

© 2024 Dupont D et al. *JAMA Network Open.*

Model	Outcome	Contrast	Timepoint	OR (95% CI)
13	Irritability	Concussion vs OI	1-month timepoint	2.81 (1.38, 5.72)
13	Irritability	Concussion vs CC	1-month timepoint	3.76 (1.98, 7.14)
13	Irritability	Concussion vs OI	3-month timepoint	1.49 (0.70, 3.19)
13	Irritability	Concussion vs CC	3-month timepoint	1.38 (0.74, 2.55)
14	Mood/Motivation	Concussion vs OI	Pre-Injury	3.48 (0.99, 12.18)
14	Mood/Motivation	Concussion vs CC	Pre-Injury	1.81 (0.69, 4.71)
14	Mood/Motivation	Concussion vs OI	ED visit	1.69 (0.82, 3.51)
14	Mood/Motivation	Concussion vs CC	ED visit	7.12 (2.64, 19.22)
14	Mood/Motivation	Concussion vs OI	10-day timepoint	1.85 (0.87, 3.93)
14	Mood/Motivation	Concussion vs CC	10-day timepoint	3.43 (1.68, 6.98)
14	Mood/Motivation	Concussion vs OI	1-month timepoint	4.49 (1.78, 11.36)
14	Mood/Motivation	Concussion vs CC	1-month timepoint	3.64 (1.70, 7.77)
14	Mood/Motivation	Concussion vs OI	3-month timepoint	1.63 (0.68, 3.90)
14	Mood/Motivation	Concussion vs CC	3-month timepoint	1.65 (0.81, 3.35)

© 2024 Dupont D et al. *JAMA Network Open.*

eFigure 1. Distribution of PCS at the Scale- and Domain-Level by Group and Time

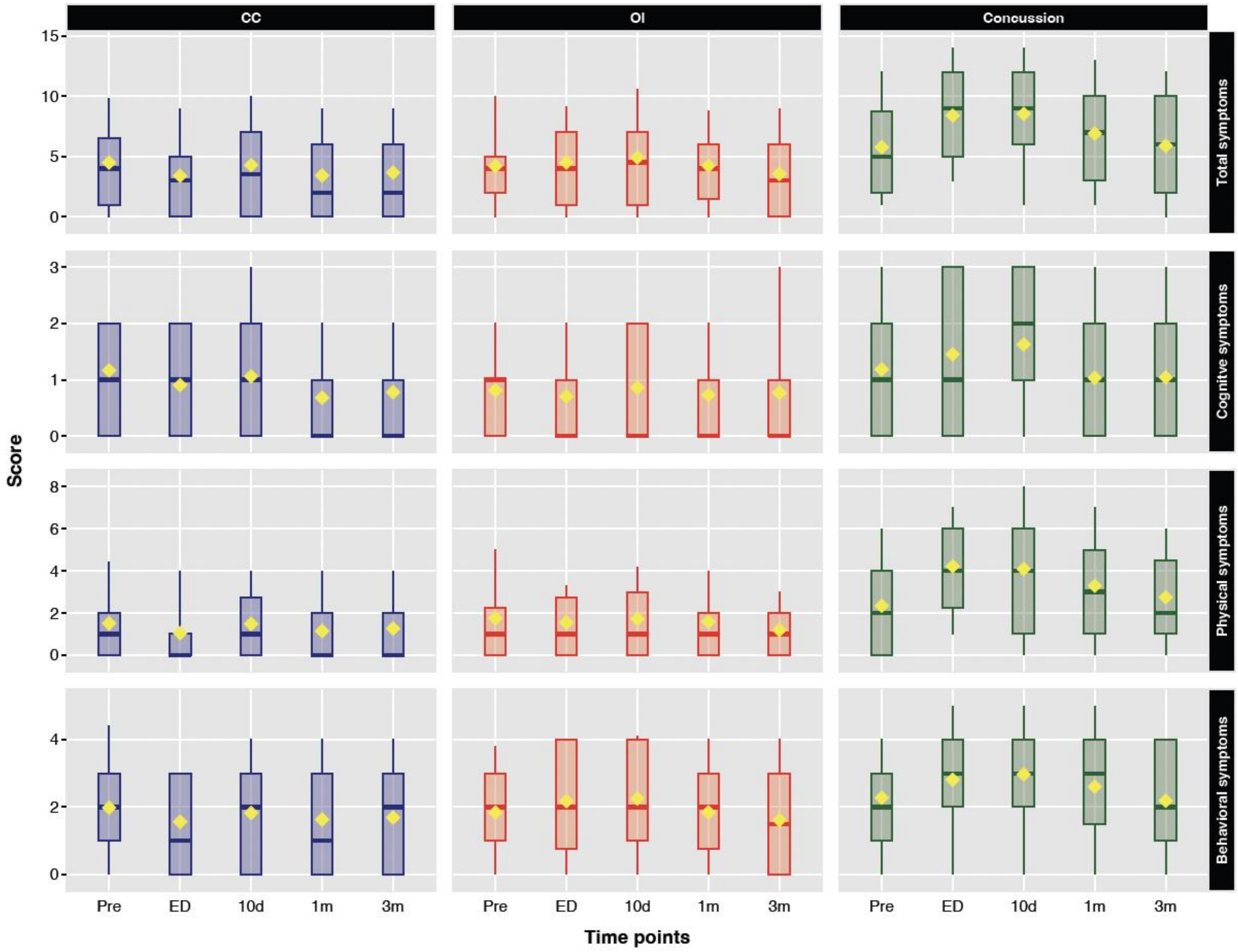

PCS score distribution at the Scale (Total symptoms) and Domain-level (Cognitive, Physical, Behavioral symptoms) by group: Community controls (CC), Orthopedic injury (OI), and Concussion. The x-axis depicts the timepoints: Pre-injury (pre), emergency department (ED), 10-days (10d), 1-month (1m), and 3-month (3m). Boxplots quantify the median (thick line), 25th and 75th quantiles (hinges), 10th and 90th quantiles (whisker ends), and the mean (diamond).

© 2024 Dupont D et al. *JAMA Network Open.*

eFigure 2. Percentage of Caregivers Endorsing Different Symptoms by Group

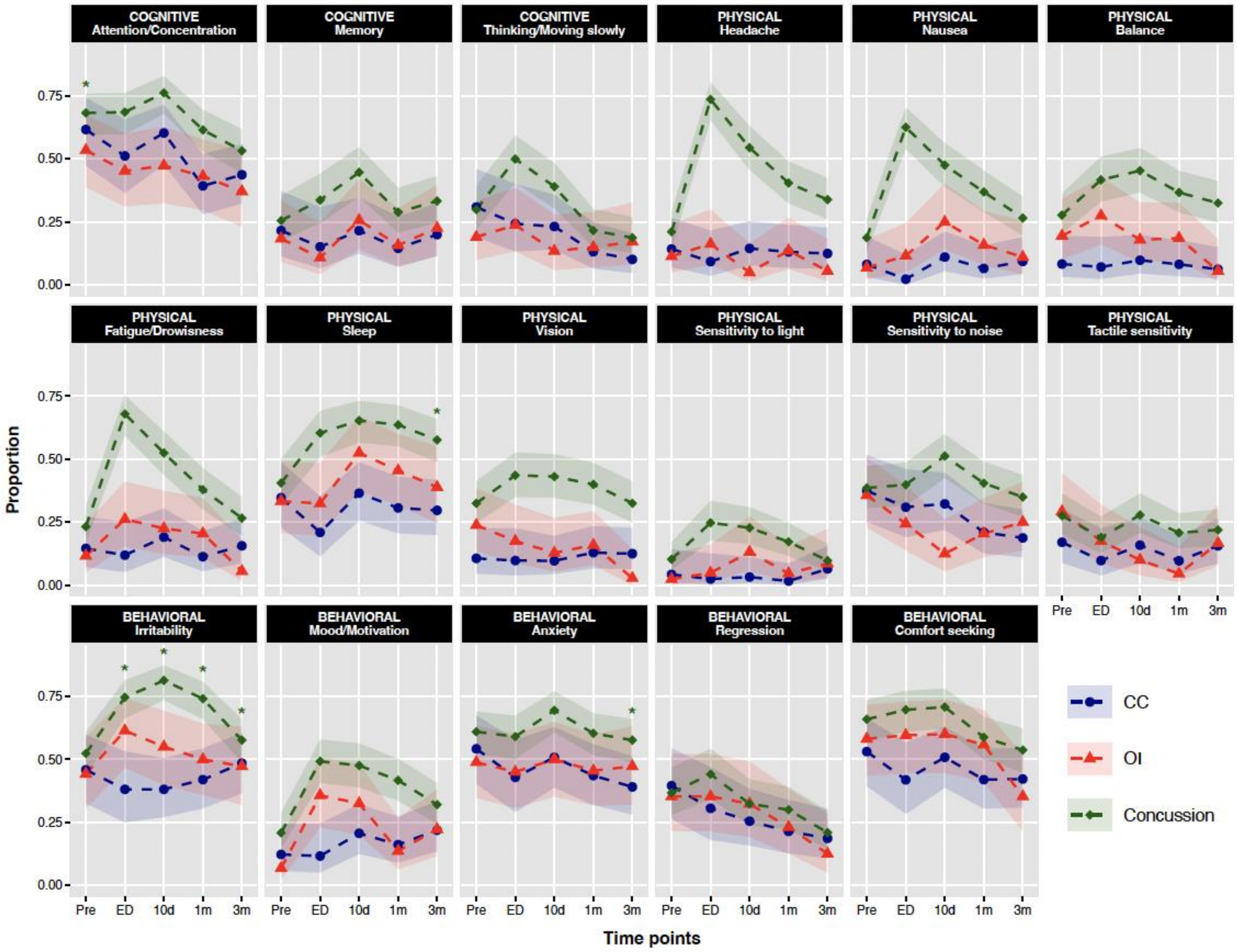

Proportion of caregivers affirming each individual symptom by group. The x-axis depicts the timepoints: Pre-injury (pre), emergency department (ED), 10-days (10d), 1-month (1m), and 3-month (3m). The asterisks indicate the symptom most frequently endorsed at that particular study timepoint across all groups.

© 2024 Dupont D et al. *JAMA Network Open.*

eFigure 3. Symptom-Level Differences Between Groups Based on Wald Test From Regression Model Fit

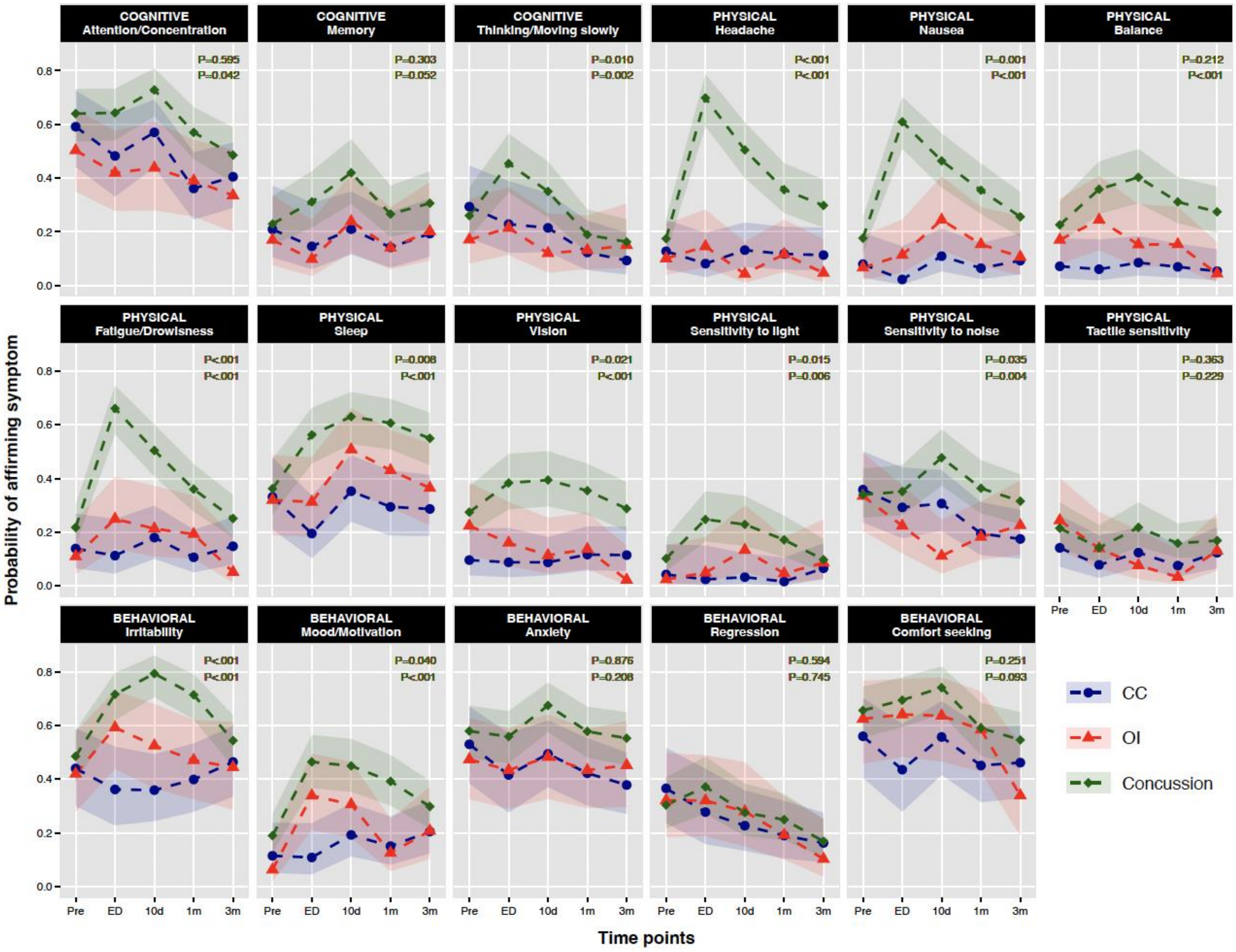

Probability of affirming each individual symptom by injury group. The x-axis depicts the timepoints: Pre-injury (pre), emergency department (ED), 10-days (10d), 1-month (1m), and 3-month (3m). Adjusted to: Age=34 months, Sex=Female; Top P values are based on Wald test for the group x time interaction and bottom P values are based on group interaction from regression model fit.

© 2024 Dupont D et al. *JAMA Network Open.*
