# Supplementary material for: Postconcussive Symptoms After Early Childhood Concussion
Source: JAMA Netw Open. 2024 Mar 21;7(3):e243182. doi: 10.1001/jamanetworkopen.2024.3182 (PMC10958232; doi:10.1001/jamanetworkopen.2024.3182)
Supplement: Supplement 2. — PERC KOALA Study Members [file jamanetwopen-e243182-s002.pdf]

Supplemental Online Content: Nonauthor Collaborators
*First name, last name, and suffix (if applicable) are required and will appear in PubMed.

*Group Name(s): PERC KOALA Study

*First Name and Middle Initial(s)	*Last Name	*Suffix (eg, Jr, III)	Academic Degrees	Institution	Location (city, state/province, country)	Role or Contribution, eg, chair, principal investigator	Group (if more than 1 Group listed in the byline) and/or Subgroup (eg, Steering Committee)
Annie	Bernier		PhD	University of Montreal	Montreal, Quebec, Canada	Co-investigator	
Catherine	Lebel		PhD	Alberta Children's Hospital	Calgary, Alberta, Canada	Co-investigator	
Ramy	El-Jalbout		MD	CHU Sainte-Justine Hospital	Montreal, Quebec, Canada	Collaborator	
Sonia	Lupien		PhD	University of Montreal	Montreal, Quebec, Canada	Co-investigator	
Louis	de Beaumont		PhD	University of Montreal	Montreal, Quebec, Canada	Co-investigator	
Mathieu	Dehaes		PhD	CHU Sainte-Justine Hospital	Montreal, Quebec, Canada	Collaborator	
Daniel	Cohen		MD	Nationwide Children's Hospital	Columbus, Ohio, Unites States	Collaborator	
Stephanie	McLellan-Lamarche		Masters	CHU Sainte-Justine Hospital	Montreal, Quebec, Canada	Study coordinator	
Lisa Marie	Langevin		PhD	Alberta Children's Hospital	Calgary, Alberta, Canada	Site coordinator	
Joanna	Mazza			Montreal Children's Hospital	Montreal, Quebec, Canada	Site coordinator	
Taylour	Aungst			Nationwide Children's Hospital	Columbus, Ohio, Unites States	Site coordinator	
