# Supplementary material for: Postconcussive Symptoms After Early Childhood Concussion
Source: JAMA Netw Open. 2024 Mar 21;7(3):e243182. doi: 10.1001/jamanetworkopen.2024.3182 (PMC10958232; doi:10.1001/jamanetworkopen.2024.3182)
Supplement: Supplement 3. — Data Sharing Statement [file jamanetwopen-e243182-s003.pdf]

Data Sharing Statement

Dupont. Postconcussive Symptoms After Early Childhood Concussion. *JAMA Netw Open*. Published March 21, 2024. doi:10.1001/jamanetworkopen.2024.3182

Data
Data available: No

Additional Information
Explanation for why data not available: The data will be shared on reasonable request to the corresponding author.
